# Genotype-phenotype correlation in Taiwanese children with diazoxide-unresponsive congenital hyperinsulinism

**DOI:** 10.3389/fendo.2023.1283907

**Published:** 2023-11-16

**Authors:** Cheng-Ting Lee, Wen-Hao Tsai, Chien-Ching Chang, Pei-Chun Chen, Cathy Shen-Jang Fann, Hsueh-Kai Chang, Shih-Yao Liu, Mu-Zon Wu, Pao-Chin Chiu, Wen-Ming Hsu, Wei-Shiung Yang, Ling-Ping Lai, Wen-Yu Tsai, Shi-Bing Yang, Pei-Lung Chen

**Affiliations:** ^1^ Department of Pediatrics, National Taiwan University Hospital and National Taiwan University College of Medicine, Taipei, Taiwan; ^2^ Graduate Institute of Clinical Medicine, National Taiwan University College of Medicine, Taipei, Taiwan; ^3^ Institute of Biomedical Sciences, Academia Sinica, Taipei, Taiwan; ^4^ Department of Physiology, National Cheng-Kung University, Tainan, Taiwan; ^5^ Department of Pathology, National Taiwan University Hospital and National Taiwan University College of Medicine, Taipei, Taiwan; ^6^ Department of Pediatrics, Kaohsiung Veterans General Hospital, Kaohsiung, Taiwan; ^7^ Department of Surgery, National Taiwan University Hospital and National Taiwan University College of Medicine, Taipei, Taiwan; ^8^ Department of Internal Medicine, National Taiwan University Hospital and National Taiwan University College of Medicine, Taipei, Taiwan; ^9^ Graduate Institute of Medical Genomics and Proteomics, National Taiwan University College of Medicine, Taipei, Taiwan; ^10^ Department of Medical Genetics, National Taiwan University Hospital, Taipei, Taiwan

**Keywords:** congenital hyperinsulinism, *ABCC8*, *KCNJ11*, *GCK*, K_ATP_ channel, founder mutation

## Abstract

**Objective:**

Congenital hyperinsulinism (CHI) is a group of clinically and genetically heterogeneous disorders characterized by dysregulated insulin secretion. The aim of the study was to elucidate genetic etiologies of Taiwanese children with the most severe diazoxide-unresponsive CHI and analyze their genotype-phenotype correlations.

**Methods:**

We combined Sanger with whole exome sequencing (WES) to analyze CHI-related genes. The allele frequency of the most common variant was estimated by single-nucleotide polymorphism haplotype analysis. The functional effects of the ATP-sensitive potassium (K_ATP_) channel variants were assessed using patch clamp recording and Western blot.

**Results:**

Nine of 13 (69%) patients with ten different pathogenic variants (7 in *ABCC8*, 2 in *KCNJ11* and 1 in *GCK*) were identified by the combined sequencing. The variant *ABCC8* p.T1042QfsX75 identified in three probands was located in a specific haplotype. Functional study revealed the human SUR1 (hSUR1)-L366F K_ATP_ channels failed to respond to intracellular MgADP and diazoxide while hSUR1-R797Q and hSUR1-R1393C K_ATP_ channels were defective in trafficking. One patient had a *de novo* dominant mutation in the *GCK* gene (p.I211F), and WES revealed mosaicism of this variant from another patient.

**Conclusion:**

Pathogenic variants in K_ATP_ channels are the most common underlying cause of diazoxide-unresponsive CHI in the Taiwanese cohort. The p.T1042QfsX75 variant in the *ABCC8* gene is highly suggestive of a founder effect. The I211F mutation in the *GCK* gene and three rare SUR1 variants associated with defective gating (p.L366F) or traffic (p.R797Q and p.R1393C) K_ATP_ channels are also associated with the diazoxide-unresponsive phenotype.

## 1 Introduction

Congenital hyperinsulinism (CHI) is a rare inherited disorder in the metabolism of glucose characterized by dysregulation in insulin secretion. Patients present with severe hypoglycemia associated with inappropriate insulin secretion. Delayed diagnosis may result in irreversible brain damage due to prolonged hypoglycemia (1). CHI has a heterogeneous genetic etiology, and pathogenic variants in *ABCC8*, *KCNJ11*, *GLUD1, GCK, HADH*, *SLC16A1*, *HNF4A*, *HNF1A*, *HK1*, and *INSR* genes have been identified to cause isolated, persistent CHI (2).

In pancreatic β cells, the ATP-sensitive potassium (K_ATP_) channel plays a critical role in coupling changes in the plasma glucose concentration to electrical excitability and insulin release (3). The K_ATP_ channel is an octameric complex consisting of four subunits of the pore-forming potassium channel subunit (Kir6.2) and four sulfonylurea receptor 1 (SUR1) subunits, which are encoded by *KCNJ11* and *ABCC8* genes, respectively. At the resting metabolic state, opening of the K_ATP_ channels hyperpolarizes the cell membrane. As circulating glucose levels rise, glucose enters the pancreatic β cells and increases the intracellular ATP concentration ([ATP]i) through the glucokinase-mediated glycolysis pathway. This elevated [ATP]i leads to the closure of K_ATP_ channels, causing the depolarization of pancreatic β cells and resulting in insulin secretion. In addition to ATP, K_ATP_ channels are modulated by different intracellular molecules, including the phospholipid PIP_2_ and magnesium (Mg)-nucleotides such as MgATP and MgADP (4–6).

CHI is classified as diffuse, focal, or atypical form depending on the pancreatic histological assessment. The diffuse form of CHI is generally associated with recessive loss-of-function variants in the *ABCC8* and *KCNJ11* genes. Patients with this condition are usually unresponsive to potassium channel openers (e.g., diazoxide), and extensive pancreatectomy is required to ameliorate hypoglycemia. Most cases of focal CHI are related to paternally inherited pathogenic variants in either *ABCC8* or *KCNJ11* and somatic loss of the maternal 11p15 region (7, 8). Although the clinical manifestations of patients with focal and diffuse CHI due to pathogenic variants in *ABCC8* or *KCNJ11* are indistinguishable, those with focal CHI can be cured by excision of the lesion (9). Atypical CHI is not easily classified as diffuse or focal form and is characterized by morphological mosaicism with hyperfunctional islets confined to discrete regions of the pancreas (10, 11). Although mosaic interstitial paternal uniparental isodisomy for chromosome 11p15.1 with an *ABCC8* gene mutation has been reported to explain one of the genetic mechanisms (10), the genetic background of most cases with atypical CHI remains unclear (11). Dominant mutations in *ABCC8* and *KCNJ11* genes, as well as disease-causing variants in other CHI-related genes, have been frequently associated with mild and diazoxide-responsive CHI (2).

In this study, we performed molecular analyses to characterize a cohort of thirteen Taiwanese children with diazoxide-unresponsive CHI. Their pathological findings were analyzed to assess genotype-phenotype correlations. Functional status of rare K_ATP_ variants in these patients was studied and the founder effect of the most prevalent pathogenic variant was elucidated.

## 2 Materials and methods

### 2.1 Patients

The diagnostic criteria for CHI were modified from those of the European Network for Research into Hyperinsulinism (12, 13): [1] laboratory blood glucose level of <50 mg/dL; [2] glucose requirement >6-8 mg/kg/min to maintain a blood glucose level of >50 mg/dL; [3] detectable insulin at the point of hypoglycemia, with raised C-peptide; [4] inappropriately low blood ketone body concentration at the time of hypoglycemia; and [5] glycemic response after glucagon administration during hypoglycemia. Patients with insulinoma, transient or syndromic hyperinsulinemic hypoglycemia were excluded. Thirteen children diagnosed with diazoxide-unresponsive CHI between 1990 and 2016 at National Taiwan University Hospital were enrolled. Non-responsiveness to diazoxide was defined as failure to keep blood glucose level >50 mg/dL in a five-day trial of oral diazoxide therapy at 15 mg/kg/day (neonates) or 10 mg/kg/day (infants) divided into three doses (14).

### 2.2 DNA isolation and genotyping

Genomic DNA was extracted using the commercially available QIAamp DNA Blood Mini Kit (QIAGEN, Germany). Direct Sanger sequencing of *ABCC8* and *KCNJ11* was performed. When no pathogenic variants were identified, we sequenced other CHI-related genes. PCR primers for the coding exons were designed using the Primer3 software (http://bioinfo.ut.ee/primer3-0.4.0/). The PCR products were sequenced using a BigDye terminator cycle sequencing kit (Applied Biosystems, Foster City, CA, USA) and analyzed by the 3130xl genetic analyzer (Applied Biosystems). The sequences were compared with published sequence data (*ABCC8*-NM_000352.3, *KCNJ11*-NM_000525.3, *GLUD1*-NM_005271.3, *GCK*-NM_000162.3, *HADH*-NM_005327.4, *SLC16A1*-NM_003051.3, *HNF4A*-NM_000457.3, *HNF1A*-NM_000545.6). The significance of the identified variants was accessed by Human Gene Mutation Database (http://www.hgmd.cf.ac.uk/ac/index.php) and ClinVar (https://www.ncbi.nlm.nih.gov/clinvar/). Possible large genomic structural variant in *ABCC8* was analyzed using the SALSA multiplex ligation-dependent probe amplification (MLPA) kit, P117 (MRC Holland, Amsterdam, Netherlands). The pathogenicity of genetic variants was classified using the American College of Medical Genetics and Genomics (ACMG) criteria (15).

Whole exome sequencing (WES) was performed for the cases if no disease-causing variants were identified by Sanger sequencing. The pipeline for variant calling and filtering analysis of WES data was performed as previously described (16). The filtered variants were queried from 35 genes involved in pancreatic islet β cell function and insulin secretion (2, 17) (**Supplementary Table 1**). The potential pathogenic variants were validated by Sanger sequencing, and segregation analysis was performed.

### 2.3 Single-nucleotide polymorphisms genotyping

Three probands carrying the c.3124_3126delACCinsCAGCCAGGAACTG variant (rs786204542) in the *ABCC8* gene and a total of 68,978 control samples were genotyped using the TWBv2 SNP array (Thermo Fisher Scientific, Inc., Santa Clara, CA, USA), a genome-wide SNP chip designed in 2017 for the Taiwanese Han population (18). After genotyping, SNPs fulfilling the following criteria were included for haplotype analysis: [1] SNPs located in the surrounding region (± 100 kb) of the *ABCC8* gene; [2] with a minor allele frequency of >0.5% (excluding the rs786204542 variant); [3] a call rate >95%; and [4] satisfying the Hardy–Weinberg equilibrium (HWE) (*P* > 0.0001) (19).

### 2.4 Haplotype estimation and statistical analysis

Two scales of chromosomal haplotype regions among the cases and controls generated from the genotyped data were tested: [1] the bilateral 50 Kb regions flanking rs786204542, and [2] the bilateral 20 SNPs [110.3 Kb upstream (rs117210015) and 62.8 Kb downstream (rs2237969)] flanking rs786204542. Haplotype frequencies were estimated using the SAS/Genetics HAPLOTYPE PROCEDURE (SAS Institute, Cary, NC, USA) and the stepwise expectation-maximization (EM) algorithm (19, 20). The probability of individual’s particular haplotype was calculated given the haplotype frequency reached the maximum likelihood estimations (MLEs) by stepwise EM algorithm. Individual haplotype pairs were included in the analysis when the probability of the event was higher than 0.7 (21). The differences in haplotype frequencies between the case and control groups were assessed using Fisher’s exact test. The permutation test was used to estimate the likelihood that the three cases carry the same rare extended haplotype containing rs786204542. To drive the empirical distribution while preserving the structure of the genotype data, we performed 100,000 or 1,000,000 replications by shuffling the case-control status and calculated permutation *P* values by comparing haplotype frequencies between cases and controls (22).

### 2.5 Electrophysiological and biochemical analysis

Human Kir6.2 (hKir6.2), and SUR1 (hSUR1) (Origene, Rockville, MD, USA) were cloned into the pcDNA3 plasmid as described previously (23, 24). Site-directed mutagenesis was performed using Phusion Flash high-fidelity DNA polymerase (Thermo Fisher Scientific, Waltham, MA, USA), and the mutations were verified by sequencing. HEK293 cells were cultured in DMEM (Thermo Fisher Scientific, Waltham, MA, USA) containing 10% FBS (Hyclone, Logan, UT, USA), and 2 mM glutamine, in a humidified atmosphere of 5% CO_2_ at 37°C. Cells were plated on poly L-ornithine-coated glass coverslips and transiently transfected with 0.2 μg of the pcDNA3 containing hKir6.2 construct and 0.8 μg of pcDNA3 containing hSUR1 construct by using PolyJet (SignaGen, Rockville, MD, USA). Cells were used 2–4 days after transfection. K_ATP_ currents were recorded by an Axopatch 700B amplifier (Molecular Device, Sunnyvale, CA, USA) and data were acquired at 10 kHz with pClamp 10 software (Molecular Device, Sunnyvale, CA, USA). For whole-cell patch clamp recording, the extracellular solution consisted of 150 mM NaCl, 10 mM HEPES, 5 mM KCl, 2 mM CaCl_2_, 1 mM MgCl_2_ and pH 7.2, adjusted with NaOH. The intracellular solution containing: 135 mM K gluconate, 15 mM KCl, 10 mM HEPES, 0.5 mM Mg_2_ATP, 1 mM Na_3_GTP, 10 mM sodium phosphocreatine, 0.05 mM EGTA and pH 7.2, adjusted with KOH. For inside-out patch clamp recording, both the pipette and bath solution contained a high-potassium solution consisting of: 150 mM KCl, 10 mM HEPES, 2 mM CaCl_2_, 1 mM MgCl_2_ and pH 7.2, adjusted with KOH. ATP (Sigma Aldrich, USA), ADP (Sigma Aldrich, USA) and diazoxide (Hello Bio, UK) were added to the bath solution as indicated. Pipettes were pulled from 1.5 mm borosilicate glass capillaries (Sutter, Novato, CA, USA). The access resistances ranged between 5 and 20 MΩ and were compensated by ~80%. All experiments were performed at room temperature (~25°C). Data were analyzed with pClamp10 software (Molecular Devices, Sunnyvale, CA, USA). Results are reported as mean ± SEM. Statistical analysis was performed using Prism 7 (GraphPad, La Jolla, CA, USA), with differences considered significant at *P* < 0.05. EC_50_ and IC_50_ of nucleotides or diazoxide on the K_ATP_ currents were calculated as described (25). For Western blot analysis, the K_ATP_ channel-transfected HEK293 cell lysate was run on SDS-PAGE and transferred to a nitrocellulose membrane. The membrane was hybridized with a rabbit anti-SUR1 antibody (#PA5-103639, Thermo Fisher, USA), then incubated with horseradish peroxidase-conjugated secondary antibody (GE Healthcare, Little Chalfont, UK), and proteins were visualized by enhanced chemiluminescence (Super Signal West Femto, Pierce, IL, USA). β-actin was used as a loading control. For structure modeling, apo- (PDB: 6JB1) (26) and MgATP/MgADP-bound K_ATP_ channels (PDB: 6C3P) (6) were used as templates and visualized by PyMOL (http://www.pymol.org/).

## 3 Results

The present study included 13 patients (eight boys and five girls) suffering from diazoxide-unresponsive CHI. Clinical data are shown in **Table 1**. Patients’ data were described previously (13), except for patients No.3 and 7. After extensive molecular analysis, pathogenic variants were identified in nine (69%) patients.

**Table 1:** Clinical characteristics and genotype profiles of 13 patients with diazoxide-unresponsive congenital hyperinsulinism.

Patient No./Sex	GA(wks) /BBW (g)	Age at onset	Max GIR (mg/kg/min)	OP/ Histology	Post-OP medication	Mutation					
						Gene	Exon/ intron	Nucleotide change	Amino acid substitution	Parental origin	ACMG classification
1/M	38/5664	<1 d	25.3	NP/Dif	–	*ABCC8*	Exon 25	c.3124_3126delACCins CAGCCAGGAACTG	p.T1042QfsX75	Maternal	Pathogenic
							Exon 35	c.4252C>T	p.R1418C	Paternal	Likely Pathogenic
2/F	36/3960	<1 d	21.0	NP/Dif	D,O	*ABCC8*	Exon 7	c.1096C>T	p.L366F	Paternal	Uncertain Significance
							Exon 19	c.2390G>A	p.R797Q	Maternal	Uncertain Significance
3/F	36/4950	<1 d	20.0	NP/Dif	D,O	*ABCC8*	Intron 7	c.1177-2A>G	Aberrant splicing	Paternal	Pathogenic
							Exon 25	c.3124_3126delACCins CAGCCAGGAACTG	p.T1042QfsX75	Maternal	Pathogenic
4/F	39/4310	<1 d	23.0	NP/Dif	O	*KCNJ11*	Exon 1	c.844G>A	p.E282K	Paternal	Uncertain Significance
5/M	38/4100	1 d	24.0	NP/Dif	D	–	–	–	–	–	–
6/M	FT/5840	2 d	14.0	NP/Dif	D	*GCK*	Exon 6	c.631A>T	p.I211F	*De novo*	Likely Pathogenic
7/M	38/3380	3 d	19.0	NP/Dif	D	*ABCC8*	Exon 25	c.3124_3126delACCins CAGCCAGGAACTG	p.T1042QfsX75	Paternal	Pathogenic
							Exon 34	c.4177 C>T	p.R1393C	Maternal	Pathogenic
8/F	39/4120	2 m	11.3	NP/Dif	O	*KCNJ11*	Exon 1	c.101G>A	p.R34H	Paternal	Likely Pathogenic
9/M	40/3900	2 m	11.6	SP/Dif	–	–	–	–	–	–	–
10/M	39/3920	3 m	15.0	EN/Fo	–	*ABCC8*	Exon 2	c.221G>A	p.R74Q	Paternal	Likely Pathogenic
11/M	38/3670	4 m	13.0	NP/Dif	–	–	–	–	–	–	–
12/F	40/4300	4 m	25.0	NP/AtDif	D,O	GCK*	Exon 6	c.631A>T	p.I211F	*De novo*	Likely Pathogenic
13/M	38/3400	8 m	8.0	NP/Dif	D	–	–	–	–	–	–

M, male; F, female; FT, full-term; d, day; m, month; max GIR, maximum glucose infusion rate; NP, near-total pancreatectomy; SP, subtotal pancreatectomy; EN, enucleation; Dif, diffuse; Fo, focal; AtDif, atypical diffuse; D, diazoxide; O, octreotide; ACMG, American College of Medical Genetics and Genomics.

*Identified as postzygotic mutation by whole exome sequencing.

### 3.1 *ABCC8* variants

Seven different pathogenic variants of the *ABCC8* gene in five of the nine (56%) patients were identified. These pathogenic variants included five missense, one indel, and one splice site variants. The indel c.3124_3126delACCinsCAGCCAGGAACTG (p.T1042QfsX75) detected in three unrelated patients was the most common pathogenic variant identified in this study. All the three patients were compound heterozygotes, with the other pathogenic variants as p.R1393C, p.R1418C, and a novel c.1177-2A>G variant. Two of these patients inherited the indel variant from their unaffected mothers, while the third patient (patient No.7) received the indel from the unaffected father. The fourth patient was compound heterozygous for p.L366F and p.R797Q in the *ABCC8* gene and inherited from her unaffected parents, respectively. All these four patients had CHI diagnosed within one week after birth. Two (Patient No.3 and 7) suffered from hypoglycemic seizure before the diagnosis was confirmed. Because of a high demand of intravenous glucose supplement and poor response to high dose diazoxide treatment, all of them received a near-total pancreatectomy. The histological assessment showed diffuse islet cell hyperplasia in these four patients. One patient (patient No.1) developed diabetes mellitus immediately after pancreatectomy. Hypoglycemia persisted after pancreatectomy in the other three patients and was controlled by diazoxide only or in combination with octreotide.

In patient No.10, a single pathogenic variant (p.R74Q), located in the *ABCC8* gene and inherited from his father, was identified. The MLPA analysis did not detect deletions in the *ABCC8* gene. This patient presented with hypoglycemic seizure at the age of three month. CHI was diagnosed at the age of five month. He had focal adenomatous hyperplasia over the body of the pancreas. Euglycemia was achieved after resection of the focal lesion in this patient when he was 6 months old.

### 3.2 *KCNJ11* variants

We observed two pathogenic missense variants of *KCNJ11* in two of the nine (22%) patients. Both patients were in the heterozygous state and no additional genetic defect was detected by MLPA analysis. The first patient carried the p.E282K pathogenic variant of paternal origin. Her paternal history revealed no hypoglycemia in infancy or childhood, but diabetes mellitus was diagnosed in the 3rd decade of life and was controlled by an oral hypoglycemic agent. This patient, born at full term with a birth weight of 4,310 gram, presented with hypoglycemic seizure on the first day after birth. The diagnosis of CHI was confirmed soon after the onset of symptom. The second heterozygous patient carried the paternally inherited p.R34H variant in *KCNJ11* along with a rare missense variant c.853C>T (p.R285W) in *ABCC8*, which was categorized as a variant of unknown significance (VUS) in ClinVar. No hypoglycemia or diabetes mellitus was reported in paternal history. This patient also had macrosomia at birth with a birth body weight of 4,120 gram. She was noted to have hypoglycemic seizure at a later age when she was two months old and then CHI was diagnosed. Both two patients received a near-total pancreatectomy due to a high demand of intravenous glucose infusion to maintain euglycemia and unresponsiveness to high dose of diazoxide. The histological examination of the pancreas in both patients showed diffuse islet cell hyperplasia. They both needed octreotide to control hypoglycemia after pancreatectomy.

### 3.3 *GCK* variants

The *de novo* p.I211F mutation of *GCK* was identified in patient No.6 (**Supplementary Figure 1A**). He was born at full term with a birth weight of 5,840 grams and had hyperinsulinemic hypoglycemia that was diagnosed at the age of two days. Intravenous glucose infusion at a rate of 14 mg/kg/min was required to maintain euglycemia. The patient showed a poor response to diazoxide, and a near-total pancreatectomy was performed at the age of one month. Hypoglycemia persisted after surgery, but this condition was controlled with diazoxide and frequent feeding.

The pathogenic variant *GCK* c. 631A>T (p.I211F) was detected in 7 of 58 (12%) reads by WES of patient No.12 (**Supplementary Figure 1C**), which was failed to be detected by Sanger sequencing (**Supplementary Figure 1B**). She was born at full term with a birth weight of 4,300 grams. Several seizure episodes were documented at the age of 4 months. Hyperinsulinemic hypoglycemia was diagnosed at the age of 11 months. The patient needed glucose infusion at a rate as high as 25 mg/kg/min to maintain euglycemia. She did not respond to a high dose of diazoxide, and a near-total pancreatectomy was performed at the age of one year. The pathohistological examination of the pancreas revealed diffuse islet changes with multiple discrete intralobular islet cell hyperplasia. After surgery, the patient maintained euglycemia through therapy with diazoxide and octreotide.

### 3.4 SNP haplotype analysis of the *ABCC8* gene

Two scales of haplotype regions containing the recurrent indel variant c.3124_3126delACC ins CAGCCAGGAACTG (rs786204542) were examined. **Supplementary Table 2** displays the list of SNPs found within the test region. The haplotype configurations, consisting of 40-SNP combinations (comprising 20 SNPs on each side flanking the rs786204542 variant) are shown in **Table 2**. The haplotype frequency of “haplotype B” in 4062 controls was 3.53%, while the frequency in these three cases was 66.67%. All three tested cases carried at least one “haplotype B” allele. Based on our statistical analysis, it is unlikely that these three patients having the same rare variant rs786204542 coincidentally carried “haplotype B” (Fisher’s exact *P* value = 2.28 × 10^−5^; permutation *P* value < 10^−6^).

**Table 2:** Estimated single nucleotide polymorphism (SNP) haplotype frequency with statistics in the region of bilateral 20 SNPs flanking the indel variant rs786204542.

Haplotype Name	Haplotype SNP Composition^a^	Affected patients (n=3)		Controls (n=4062)		Fisher’s exact test *P*-value^b^	Permutation *P*-value^c^
		Count	Percent (%)	Count	Percent (%)		
**Haplotype A**	2-2-2-2-2-2-2-2-2-2-2-2-2-2-2-2-2-2-2-2-2-2-2-2-2-2-2-2-2-2-2-2-1-2-2-2-2-2-2-1	1	16.67	644	7.93	0.3911	
Haplotype B*	2-2-2-2-2-2-2-2-2-2-2-2-2-2-2-2-2-2-2-2-2-2-2-2-2-2-2-2-2-2-2-2-2-2-1-2-2-2-2-2	4	66.67	287	3.53	2.28x10^-5^	< 10^-6^
**Haplotype C**	2-2-2-2-2-2-2-2-2-2-2-2-2-2-2-2-2-2-1-2-1-2-1-2-1-1-1-2-2-2-2-2-2-2-2-2-2-2-2-1	1	16.67	264	3.25	0.18036	
**Haplotype D**	2-2-2-2-2-2-2-2-2-2-2-2-2-2-2-2-2-2-2-2-2-2-2-2-2-2-2-2-2-2-2-2-2-2-2-2-2-2-2-1	0	0	155	1.91		
**Haplotype E**	2-2-2-1-1-2-1-2-2-2-1-1-1-2-2-2-2-1-2-2-2-2-2-2-2-2-2-2-2-2-2-2-2-2-2-1-2-2-2-2	0	0	62	0.76		
**Haplotype F**	2-2-2-2-2-2-2-2-2-2-2-2-2-2-2-2-2-2-2-2-2-2-2-2-2-2-1-2-2-2-2-2-2-2-2-2-2-2-2-2	0	0	49	0.60		
**Haplotype G**	2-2-2-2-1-2-2-1-2-2-2-2-2-2-1-2-2-2-2-2-2-2-2-2-2-2-2-2-2-2-2-2-1-2-2-2-2-2-2-1	0	0	48	0.59		
**Haplotype H**	2-2-2-2-1-2-2-1-2-2-2-2-2-2-1-2-2-2-2-2-2-2-2-2-2-2-2-2-2-2-2-2-2-2-2-2-2-1-2-2	0	0	13	0.16		
**Haplotype I**	2-2-2-2-1-2-2-1-2-2-2-2-2-2-1-2-2-2-2-2-2-2-2-2-2-2-2-2-2-2-2-2-2-2-1-2-2-2-2-2	0	0	7	0.09		
**Haplotype J**	2-2-2-1-1-2-1-2-2-2-2-2-2-2-2-2-2-2-1-1-2-2-1-2-2-2-1-2-1-2-2-2-2-2-2-2-2-1-2-2	0	0	2	0.02		

^a^SNP allele “2”: major allele; SNP allele “1”: minor allele.

^b^Comparisons of haplotype frequencies between affected patients and controls using Fisher’s exact test.

^c^Comparison of frequencies of haplotype B between affected patients and controls using permutation test, permutation time=1000000.

*Common haplotype among affected patients.

The 100 Kb-SNP haplotypes, encompassing SNPs within bilateral 50 Kb regions flanking rs786204542 (a subset of the aforementioned 40-SNP haplotypes), are shown in **Table 3**. All these three tested cases were found to carry at least one “haplotype M” allele, which share the same allele composition as “haplotype B” within the overlapping region of the 40-SNP scenario. Similarly, the statistical analysis strongly disfavored the possibility of chance occurrences (Fisher’s exact *P* value = 1.08 × 10^−4^; permutation *P* value < 10^−5^).

**Table 3:** Estimated single nucleotide polymorphism (SNP) haplotype frequency with statistics in the region of bilateral 50 kb flanking the indel variant rs786204542.

Haplotype Name	Haplotype SNP Composition^a^	Affected patients (n=3)		Controls (n=4050)		Fisher’s exact test *P*-value^b^	Permutation *P*-value^c^
		Count	Percent (%)	Count	Percent (%)		
**Haplotype K**	2-2-2-2-2-2-2-2-2-2-2-2-2-2-2-2-2-2-2-2-2-2-2-1-2-2-2	1	16.67	879	10.85	0.4982	
**Haplotype L**	2-2-2-2-2-2-2-2-2-2-2-2-2-2-2-2-2-2-2-2-2-2-2-2-2-2-2	0	0	541	6.68		
Haplotype M*	2-2-2-2-2-2-2-2-2-2-2-2-2-2-2-2-2-2-2-2-2-2-2-2-2-1-2	4	66.67	426	5.26	1.08x10^-4^	< 10^-5^
**Haplotype N**	2-2-2-2-2-2-2-2-2-1-2-1-2-1-2-1-1-1-2-2-2-2-2-2-2-2-2	1	16.67	422	5.21	0.2751	
**Haplotype O**	2-1-1-1-2-2-2-2-1-2-2-2-2-2-2-2-2-2-2-2-2-2-2-2-2-2-1	0	0	60	0.74		
**Haplotype P**	2-2-2-2-2-2-2-2-2-1-1-2-2-1-2-2-2-1-2-1-2-2-2-2-2-2-2	0	0	54	0.67		
**Haplotype Q**	2-2-2-2-2-1-2-2-2-2-2-2-2-2-2-2-2-2-2-2-2-2-2-2-2-2-2	0	0	39	0.48		
**Haplotype R**	2-2-2-2-2-1-2-2-2-1-2-1-2-1-2-1-1-1-2-2-2-2-2-2-2-2-2	0	0	25	0.31		
**Haplotype S**	2-2-2-2-2-1-2-2-2-2-2-2-2-2-2-2-2-1-2-2-2-2-2-2-2-2-2	0	0	2	0.02		

^a^SNP allele “2”: major allele; SNP allele “1”: minor allele.

^b^Comparisons of haplotype frequencies between affected patients and controls using Fisher’s exact test.

^c^Comparison of frequencies of haplotype M between affected offspring and controls using permutation test, permutation time=100000.

*Common haplotype among affected patients.

These findings provide robust evidence supporting the presence of rs786204542 within haplotype B/haplotype M, suggestive of a founder effect.

### 3.5 Functional characterization of K_ATP_ channel variants

We performed whole-cell recording to examine membrane currents. Weak-inwardly rectifying potassium current was observed in HEK293 cells transfected with wild-type K_ATP_ channels and dialyzed with MgATP (0.5 mM). The application of 300 µM diazoxide enhanced the current, whereas 5 µM glibenclamide inhibited it (**Supplementary Figure 2B**). In contrast, cells transfected with hSUR1-R797Q and hSUR1-R1393C K_ATP_ channels showed significantly reduced potassium currents and did not respond to K_ATP_ channel modulators (**Supplementary Figures 2A, C, D**).

**Figure 1:**
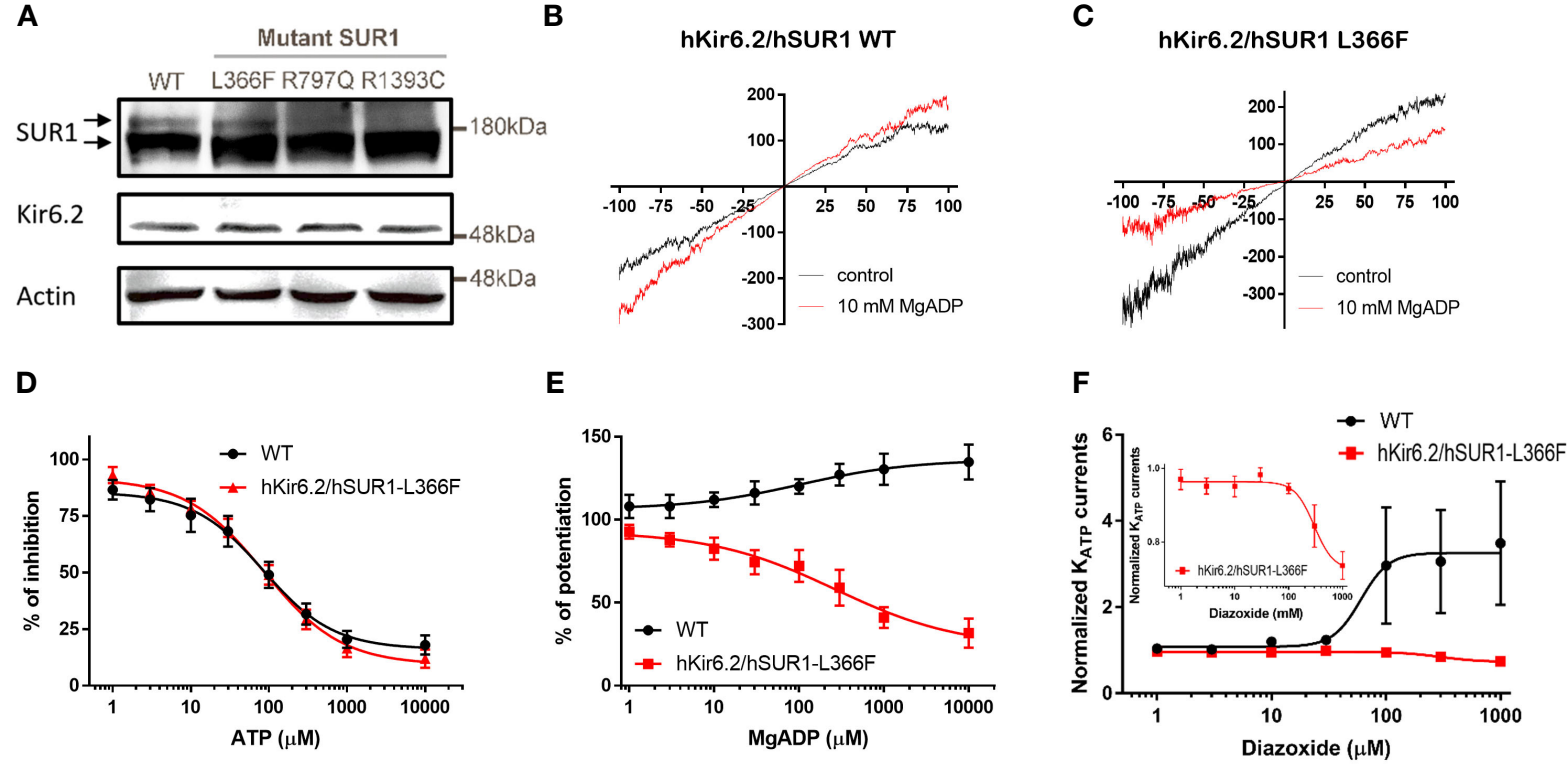
**(A)** Western blots show total SUR1 levels in HEK293 cells. SUR1 signals exhibit an upper band that corresponds to a fully glycosylated K_ATP_ channel (upper arrow), while the lower band represents immature K_ATP_ channels that are still stuck intracellularly (lower arrow) in wild-type (WT) SUR1. The upper bands are visible in HEK293 cells transfected with WT hSUR1 and hSUR1-L366F but absent in HEK293 cells transfected with hSUR1-R797Q and hSUR1-R1393C. β-actin serves as the loading control. **(B, C)** Representative voltage-clamp recording traces from inside-out patches containing K_ATP_ channels. Adding 10 mM MgADP (red traces) to the intracellular side potentiated WT hsSUR1 **(B)** but inhibited hSUR1-L366F **(C)** containing K_ATP_ currents. **(D)** WT and hSUR1-L366F containing K_ATP_ channels exhibited comparable sensitivity to the intracellular ATP (WT: IC_50 = _85.4 µM, Hill slope = 0.97, n = 4; hSUR1-L366F: IC_50 = _86.1 µM, Hill slope = 0.87, n = 6–8). **(E)** Intracellular MgADP potentiated WT K_ATP_ channels but inhibited hSUR1-L366F K_ATP_ (WT: EC_50 = _102 µM, Hill slope = 0.65, n = 4–5; hSUR1-L366F: IC_50 = _268 µM, Hill slope = 0.59, n = 3). **(F)** Diazoxide potentiates WT K_ATP_ Currents (EC_50 = _60.1 μM, n = 5) but inhibits hSUR1-L366F K_ATP_ currents (IC_50 = _297.7 μM, n = 4). The insert displays an enlarged dose-response curve of diazoxide on hSUR1-L366F K_ATP_ currents to highlight the inhibitory effect of diazoxide.

It has been reported that the cell membrane K_ATP_ channel extensively glycosylated upon maturation is evidenced by an upper band on the Western blot (27). We observed this distinctive banding pattern in cells transfected with wild-type and hSUR1-L366F K_ATP_ channels, but not in cells with hSUR1-R797Q or hSUR1-R1393C variants (**Figure 1A**). These results suggest that hSUR1-R797Q and hSUR1-R1393C mutant channels show trafficking defects.

**Figure 2:**
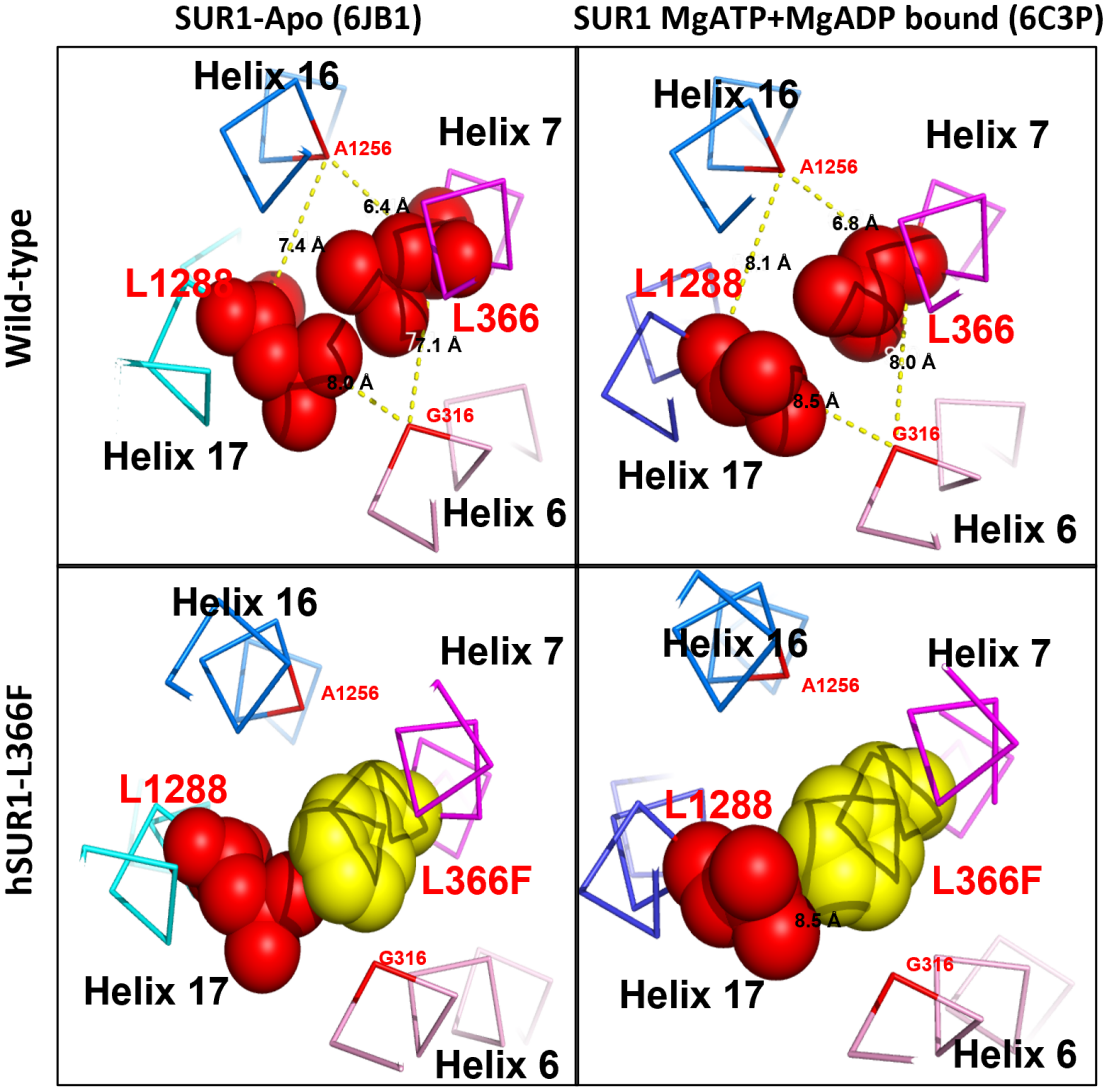
Structural prediction of SUR1 L366 and its interacting partner L1288 of K_ATP_ channel. In wild-type K_ATP_ channel, SUR1 L366 (red) could contact freely with its partner L1288 (red) via hydrophobic interaction in both Apo (PDB: 6JB1) and MgATP + MgADP bound form (PDB:6C3P) (upper panel). In contrast, in the hSUR1-L366F mutant K_ATP_ channel, the bulky phenylalanine (yellow) produces steric clashes against L1288 and consequently prevents the MgADP-induced conformational changes of SUR1 (lower panel).

The functional change in hSUR1-L366F K_ATP_ mutant channels was further examined using the inside-out patch clamp configuration technique. We found that HEK293 cells transfected with wild-type and mutant hSUR1-L366F K_ATP_ channels showed a typical K_ATP_ current that reversed at around 0 mV, the value of the potassium equilibrium potential under our experimental conditions (**Figures 1B, C**). A similar sensitivity to different intracellular ATP concentrations was observed in both wild-type and mutant hSUR1-L366F K_ATP_ channels (**Figure 1D**). However, hSUR1-L366F K_ATP_ channels were inhibited rather than potentiated by MgADP (**Figures 1C, E**), and more strikingly, diazoxide inhibited rather than activated hSUR1-L366F K_ATP_ channels (**Figure 1F**). These results suggest that hSUR1-L366F K_ATP_ channels have a gating defect. Leu366 is positioned at the interface between transmembrane domain 1 (TMD1) and transmembrane domain 2 (TMD2). When MgADP binds to the nucleotide binding domains formed along with the TMD1-TMD2 interface, it triggers conformational changes in SUR1 that subsequently activate the channel gate located at Kir6.2 (28). However, our structural modeling reveals that substituting Leu366 with the bulkier phenylalanine generates steric clashes with SUR1-L1288, thereby preventing the MgADP-induced conformational changes within the channel structure, as depicted in **Figure 2**.

## 4 Discussion

Two large-scale studies have reported that patients with CHI have a disease-causing variant detection rate ranging from 45.3% to 79%, and the detection rate of diazoxide-unresponsive CHI was approximately 90% (29, 30). In the present study, we identified pathogenic variants in nine of the 13 (69%) diazoxide-unresponsive patients with CHI. Patients with pathogenic variants in *ABCC8* and *KCNJ11* genes accounted for 78% (7/9) of variants detected. Our data confirm that pathogenic variants in the K_ATP_ channel-encoding genes are the most common underlying cause of diazoxide-unresponsive CHI in the Taiwanese patients studied, which is in accordance with that observed in cohorts of different ethnic backgrounds (29–35).

The indel c.3124_3126delACCinsCAGCCAGGAACTG (p.T1042QfsX75) of the *ABCC8* gene is a frameshift variant that results in a truncated SUR1 protein, which is believed to impair protein function (36). The p.T1042QfsX75 variant has been repeatedly detected in Chinese cohorts with CHI (32, 33, 37, 38), especially in patients from southern China (32, 33). In the present study, the detection of p.T1042QfsX75 in three unrelated patients suggests that this variant is a hotspot, and haplotype analysis supported that it is a founder variant in Taiwan. This may also explain the findings in southern China. One patient was compound heterozygous for the p.T1042QfsX75 indel variant and a novel *ABCC8* splice site variant, c.1177-2A>G. This splice site variant is predicted to disrupt the acceptor splice site of intron 7, resulting in the dysfunction of the protein. The other two patients were compound heterozygous for the indel and two different missense variants of the *ABCC8* gene, p.R1393C and p.R1418C, respectively. The SUR1 protein contains two nucleotide-binding domains (NBD1 and NBD2). Their dimerization is required for Mg^2+^-dependent ATP hydrolysis (4, 39). These NBD domains are also required for channel regulation by MgADP and diazoxide (40, 41). Although the Arg1393 residue located in NBD2 is presumably involved in K_ATP_ channel gating, it has been reported that the p.R1393H causes a trafficking defect because mutant channels are retained in the trans-Golgi network (42). Our functional study confirmed that cysteine substitution at this position impairs the trafficking of the mutant channel to the surface of the plasma membrane. The *ABCC8* missense variant p.R1418C in a compound heterozygous state with a deletion of *ABCC8* exon 3 has been reported in a patient of diazoxide-unresponsive diffuse CHI (43). This missense variant is also located in NBD2 of SUR1. Although we did not evaluate the functional change of this variant, there is evidence showed histidine substitution at the Arg1418 residue impaired channel activity by confining the channel to the endoplasmic reticulum (ER) (44). The cysteine substitution at the same position is presumed to have a deleterious effect on channel function as well. Further study should be done to elucidate the underlying mechanism of the p.R1418C variant.

One patient was compound heterozygous for p.L366F and p.R797Q variants of the *ABCC8* gene. The p.L366F has been reported as a diazoxide-unresponsive dominant variant with phenotypes varying from asymptomatic to CHI or diabetes within family members (45). Our *in vitro* functional assays showed that hSUR1-L366F K_ATP_ mutant channels are inhibited rather than activated by intracellular MgADP, and the reversed effect of MgADP on K_ATP_ channel gating may negatively affect glucose-stimulated insulin secretion (GSIS). Under normal conditions, lowering plasma glucose would decrease the intracellular ATP/ADP ratio and activate K_ATP_ channels that silence beta cells and cease insulin secretion. In contrast, hSUR1-L366F K_ATP_ mutant channels can be inhibited by both ATP and ADP, remaining mostly closed even at low blood glucose levels, leading to altered insulin secretion. We observed that hSUR1-L366F K_ATP_ mutant channels are insensitive to diazoxide, supporting the clinical observation that patients with CHI carrying this variant show a diazoxide-unresponsive phenotype. The Leu366 residue is located in the first transmembrane domain of SUR1, above the sulfonylurea binding pocket (28). Our structural modeling indicated that Leu366 forms a hydrophobic interaction with Leu1288. A substitution of leucine to phenylalanine at this position would induce steric clashes with Leu1288, preventing conformation changes of SUR1 that propagate the MgADP signal from NBDs (6). Regarding the p.R797Q variant, it has been reported that p.R797W is a recessive pathogenic variant detected in patients with CHI (29). Our functional study demonstrated that glutamine substitution at this site impairs K_ATP_ channel trafficking.

In this study, three of seven (43%) patients carrying K_ATP_ variants were inherited from the paternal monoallelic mutations. The remaining four patients had biallelic pathogenic variants of the *ABCC8* gene and invariably exhibited a diazoxide-unresponsive diffuse CHI phenotype. Patients carrying paternally inherited monoallelic pathogenic variants in *ABCC8* or *KCNJ11* gene showed different clinical manifestations (46). A patient with the recessive p.R74Q variant in the *ABCC8* gene had focal CHI, which was cured after the excision of the pancreatic lesion. The histological assessment of two patients with paternally inherited heterozygous p.R34H and p.E282K variants in the *KCNJ11* gene revealed diffuse islet changes. A compound heterozygote of one missense variant p.R34H and one single-base deletion variant in *KCNJ11* has been reported in a patient with severe diazoxide-unresponsive CHI (34). It is less likely that the p.R34H variant in our patient acts in a dominant negative manner because the paternal carrier was clinically asymptomatic. As for the p.E282K variant, there is evidence showed that this variant abrogates the exit signal and prevents the ER export and surface expression of the channel when expressed alone. When co-expressed, the mutant subunit was able to associate with the wild-type Kir6.2 and form functional channels (47). Although a dominant effect on K_ATP_ channel function of this variant was suspected as revealed by the history of early onset type 2 diabetes mellitus in the paternal carrier, it suggests that the p.E282K variant acts recessively in our patient to cause loss of function of K_ATP_ channel and severe diazoxide-unresponsive phenotype. Patients with paternally acquired monoallelic recessive variants in the *KCNJ11* gene showed severe diffuse CHI, likely due to the unsuccessful attempts to detect cryptic pathogenic variants by current sequencing methods. Alternatively, patients may show an atypical diffuse form due to mosaic interstitial paternal uniparental isodisomy of chromosome 11p15.1 region (10).

Dominant gain-of-function mutations in the *GCK* gene are considered a rare cause of CHI, showing a detection rate lower than 1.7% (29–33, 35). Patients with CHI caused by mutations in the *GCK* gene exhibit intact K_ATP_ channels, supporting that potassium channel openers are effective for controlling hyperinsulinemic hypoglycemia (48), contrasting to those observed in our patients. The p. I211F mutation in the *GCK* gene led to a 12-fold higher *k*_cat_/*K*_0.5,glucose_ value than that of the wild-type glucokinase, resulting in a very low threshold for GSIS (49). Before the current study, a case of atypical diffuse CHI with a somatic mosaic p.I211F mutation in *GCK* has been reported (11, 50). Unlike our patient 6 who is heterozygous for this mutation, both the two patients with the somatic p.I211F mutation in *GCK* were less overweight at birth and exhibited a more insidious onset of hypoglycemia after the neonatal period. However, these two patients eventually became diazoxide-unresponsive and needed pancreatectomy to control hypoglycemia. We speculate that the extent of β cell involvement of the mutant enzyme correlates with the severity of the initial clinical presentation in patients carrying p.I211F mutation in *GCK*, and all patients with this pathogenic variant are eventually prone to respond poorly to diazoxide treatment due to the highly active nature of the mutant enzyme. Our finding also supports somatic mutations in the *GCK* gene is a rare but important cause of atypical CHI.

In conclusion, we found that pathogenic variants in the K_ATP_ channel were the most common underlying cause of diazoxide-unresponsive CHI in thirteen Taiwanese children. We demonstrated that the high frequency of the p.T1042QfsX75 variant in the *ABCC8* gene is likely due to a founder effect in Taiwan. Our study demonstrated that patients carrying three rare SUR1 mutants with diazoxide-unresponsive phenotype are due to K_ATP_ channel gating defect (p.L366F) or trafficking defect (p.R797Q and p.R1393C). Our study also confirmed that the p.I211F variant of the *GCK* gene results in diazoxide-unresponsive CHI. Additional management, including near-total pancreatectomy, may be required to maintain euglycemia in patients with the p.I211F variant.

## Data availability statement

The data presented in the study are deposited in the DNA Data Bank of Japan (DDBJ) repository, accession number LC785398, LC785399, LC785400, LC785401, LC785402, LC785403, LC785404, LC785405, LC785406, LC785407.

## Ethics statement

The studies involving humans were approved by Research Ethics Committee of the National Taiwan University Hospital. The studies were conducted in accordance with the local legislation and institutional requirements. Written informed consent for participation in this study was provided by the participants’ legal guardians/next of kin.

## Author contributions

C-TL: Funding acquisition, Investigation, Writing – original draft, Writing – review & editing. W-HT: Investigation, Visualization, Writing – review & editing. C-CC: Formal analysis, Writing – original draft, Writing – review & editing. P-CChen: Investigation, Writing – review & editing. CF: Formal analysis, Writing – review & editing. H-KC: Investigation, Visualization, Writing – review & editing. S-YL: Resources, Writing – review & editing. M-ZW: Investigation, Writing – review & editing. P-CChiu: Resources, Writing – review & editing. W-MH: Resources, Writing – review & editing. W-SY: Conceptualization, Supervision, Writing – review & editing. L-PL: Supervision, Writing – review & editing. W-YT: Conceptualization, Supervision, Writing – review & editing. S-BY: Conceptualization, Funding acquisition, Writing – original draft, Writing – review & editing. P-LC: Conceptualization, Supervision, Writing – review & editing.

## Supplementary material

Supplementary Figure 1 Sequencing results of mutation found in the *GCK* gene.

Supplementary Figure 2 Whole-cell patch clamp recordings of HEK293 cells transfected with hSUR1-R797Q and hSUR1-R1393C K_ATP_ channels.
