# Supplementary material for: Genotype-phenotype correlation in Taiwanese children with diazoxide-unresponsive congenital hyperinsulinism
Source: Front Endocrinol (Lausanne). 2023 Nov 16;14:1283907. doi: 10.3389/fendo.2023.1283907 (PMC10687152; doi:10.3389/fendo.2023.1283907)
Supplement: Supplementary Figure 1 — Sequencing results of mutation found in the GCK gene. [file DataSheet_1.docx]

**Supplementary Table 1** The list of genes of interest for congenital hyperinsulinemia

| *ABCC8, APPL1, CEL, EIF2AK3, FOXP3, GATA4, GATA6, GCK, GLIS3, GLUD1, HADH, HK1, HNF1A, HNF1B, HNF4A, IER3IP1, INS, INSR, KCNJ11, MNX1, NEUROD1, NEUROG3, NKX2-2, PAX4, PAX6, PDX1, PGM1, PLAGL1, PTF1A, RFX6, SLC2A2, SLC16A1 SLC19A2, WFS1, ZFP57* |
| --- |

**Supplementary Table 2** The list of single nucleotide polymorphisms over the two tested chromosome regions

| **Order** | **dbSNP_RS_ID** | **Physical position**  **on Chr11**  **(GRCh38)** | **Bilateral 50 kb flanking rs786204542** | **Bilateral 20 SNPs flanking rs786204542** |
| --- | --- | --- | --- | --- |
| **1** | rs1330 | 17294482 |  |  |
| **2** | rs756249891 | 17295362 |  |  |
| **3** | rs117210015 | 17296601 |  | 20 |
| **4** | rs9645621 | 17311651 |  | 19 |
| **5** | rs114419762 | 17314598 |  | 18 |
| **6** | rs757081 | 17330136 |  | 17 |
| **7** | rs34471145 | 17330326 |  | 16 |
| **8** | rs145430884 | 17335276 |  | 15 |
| **9** | rs2051773 | 17343662 |  | 14 |
| **10** | rs16933984 | 17350801 |  | 13 |
| **11** | rs147549380 | 17355117 |  | 12 |
| **12** | rs58601261 | 17381929 | 11 | 11 |
| **13** | rs1557765 | 17382092 | 10 | 10 |
| **14** | rs5215 | 17387083 | 9 | 9 |
| **15** | rs5219 | 17388025 | 8 | 8 |
| **16** | rs8192690 | 17393023 | 7 | 7 |
| **17** | rs739689 | 17395957 | 6 | 6 |
| **18** | rs4148645 | 17396558 | 5 | 5 |
| **19** | rs200563930 | 17397205 | 4 | 4 |
| **20** | rs2074311 | 17400313 | 3 | 3 |
| **21** | rs739688 | 17406010 | 2 | 2 |
| **22** | rs2077654 | 17406354 | 1 | 1 |
| **23** | **rs786204542** | **17406924** |  |  |
| **24** | rs2237992 | 17409386 | 1 | 1 |
| **25** | rs1805036 | 17412737 | 2 | 2 |
| **26** | rs2237991 | 17418682 | 3 | 3 |
| **27** | rs76035545 | 17420146 | 4 | 4 |
| **28** | rs4757517 | 17420281 | 5 | 5 |
| **29** | rs2237989 | 17421198 | 6 | 6 |
| **30** | rs916827 | 17423479 | 7 | 7 |
| **31** | rs75866475 | 17429916 | 8 | 8 |
| **32** | rs73423065 | 17430438 | 9 | 9 |
| **33** | rs4148619 | 17430953 | 10 | 10 |
| **34** | rs873314 | 17441311 | 11 | 11 |
| **35** | rs117529620 | 17445602 | 12 | 12 |
| **36** | rs11821723 | 17445790 | 13 | 13 |
| **37** | rs117663461 | 17449352 | 14 | 14 |
| **38** | rs2283260 | 17451769 | 15 | 15 |
| **39** | rs4148613 | 17454913 | 16 | 16 |
| **40** | rs185040406 | 17460675 |  | 17 |
| **41** | rs79446995 | 17461033 |  | 18 |
| **42** | rs146917004 | 17465715 |  | 19 |
| **43** | rs2237969 | 17469789 |  | 20 |
| **44** | rs80356634 | 17474961 |  |  |
| **45** | rs1048099 | 17474969 |  |  |
| **46** | rs34731885 | 17477837 |  |  |

**Supplementary Figure 1.** Sequencing results of mutation found in the *GCK* gene. (A) Sanger sequencing revealed heterozygous *GCK* p.I211F mutation in patient 6. (B) The forward and reverse Sanger sequencing results of patient 12 showed a slightly elevated mutant allele peak that was viewed as background noise in the initial mutational analysis. (C) Integrative Genomics Viewer output of the whole exome sequencing (WES) results revealed low levels of the *GCK* c.631A>T (p.I211F) variant in the peripheral blood DNA in patient 12.


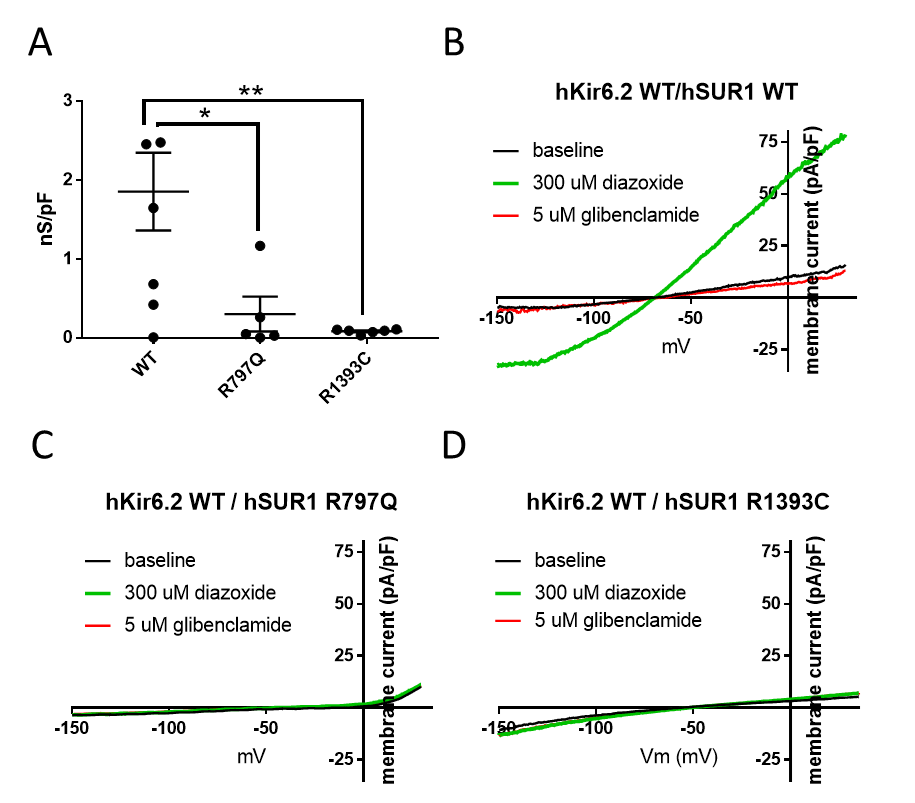


**Supplementary Figure 2.** Whole-cell patch clamp recordings of HEK293 cells transfected with hSUR1-R797Q and hSUR1-R1393C K_ATP_ channels. (A) Summary of the whole-cell K_ATP_ channel conductance normalized to the cell capacitance. HEK293 cells transfected with human SUR1 (hSUR1)-R797Q (n = 5) or hSUR1-R1393C (n = 6) had much reduced K_ATP_ conductance (**P* < 0.05, ***P* < 0.01 unpaired t-test, comparing to wild-type (WT) K_ATP_ channels (n = 6)). K_ATP_ currents were induced by voltage ramps (0.5 V s^−1^). (B) A representative whole-cell WT K_ATP_ current (black). As predicted, the K_ATP_ current was augmented by 300 µM K_ATP_ diazoxide (green) and inhibited by 5 µM glibenclamide (red). The whole-cell currents in the presence of glibenclamide served as the background reference currents for subtraction. Whole-cell recordings of HEK293 cells transfected with hSUR1-R797Q (C) or hSUR1-R1393C (D) exhibited minuscule K_ATP_ current and neither 300 µM diazoxide or 5 µM glibenclamide had an effect on the K_ATP_ current of these mutants.
